# Anoectochilusroxburghii extract down-regulates METTL3 to reduce RNA m^6^A on WNT pathway and inhibit melanoma cancer cell metastasis

**DOI:** 10.7150/jca.92212

**Published:** 2024-08-13

**Authors:** Yingyi Ye, Yazhi Tian, Jinchun Chen, Jingjun Zhao

**Affiliations:** 1 School of Medicine, Tongji University, Shanghai, 200092, China.; 2 Dermatology department of Ningbo No.2 Hospital of Zhejiang Province, Ningbo, 315200, China.; 3 Laboratory of Diagnosis and Treatment of Digestive System Tumors of Zhejiang Province, Ningbo, 315200, China.; 4 Department of Dermatology, Xinhua Hospital, School of Medicine, Shanghai Jiao Tong University, Shanghai, China.

## Abstract

Characterized by high invasiveness and expansive growth, melanoma progression is intricately regulated, with RNA m6A modifications playing a pivotal role. Among these regulators, METTL3 modulates the migratory capabilities of melanoma cells, specifically by modifying the RNA m6A of UCK2. The extract of *Anoectochilusroxburghii*, a plant known for its broad-spectrum antitumour properties, has been shown to effectively curb the invasion and migration of melanoma cells and impede tumour growth. The primary mechanism involves the downregulation of METTL3 expression. By doing so, the *Anoectochilus roxburghii* extract reduces the m6A level on the mRNA of UCK2, accelerating UCK2 mRNA degradation and, consequently, decreasing UCK2 expression. This process, in turn, suppresses gene expression within the Wnt/β-catenin pathway, contributing to its antimelanoma effects.

## Introduction

Melanoma, notoriously known for its invasive growth and resistance to therapy, is one of the deadliest types of tumours^1^-^3^. The incidence rate of melanoma is steadily increasing, growing at a rate of 3%-5% annually, and it is increasingly affecting younger populations^4^-^6^. While patients with early-stage melanoma generally have a high cure rate, those diagnosed with advanced melanoma face a grim prognosis and a low survival rate^7^.

Plant extracts, which are laden with bioactive substances, have shown promising therapeutic potential for treating melanoma and have demonstrated antitumour, antioxidant, and immunomodulatory effects^8^-^10^. Their appeal is further heightened by their minimal side effects, low toxicity, and dual utility as both medicine and food^11^. These findings underscore the importance of exploring the role of plant polysaccharides in melanoma regulation, which could pave the way for effective treatments for preventing tumour progression.

*Anoectochilusroxburghii* (AR), a member of the Orchidaceae family, is widely distributed throughout China and other Asian countries. It is known for its antitumour, immunostimulatory, and antihyperglycaemic properties^12^-^14^. Previous research has revealed that alcohol extracts of AR can significantly suppress tyrosinase activity, thereby inhibiting melanogenesis in zebrafish embryos^15^. However, the potential molecular interplay between AR extracts and melanoma migration remains an area worthy of investigation.

As one of the most important posttranscriptional mRNA modifications in eukaryotes, N^6^-methyladenine (m^6^A) is modified to recognize and regulate chemical modifications in RNA, including the m^6^A methyltransferase complex, demethylases, and m^6^A-binding proteins^16^, ^17^. m^6^A modification has received increasing attention because of its function in the occurrence and development of melanoma, such as its role in cellular differentiation^18^, ^19^. The abnormal expression of m^6^A-related proteins can lead to the proliferation, metastasis, invasion and drug resistance of melanoma cells^20^. The m6A methyltransferase METTL3 is upregulated in human melanoma tissue, which activates matrix metalloproteinase (MMP) 2, promotes the expression of c-met and p-Akt proteins, leads to the formation of melanoma cell colonies, and causes melanoma cell invasion^21^. The methyltransferase FTO is a primary factor in melanoma. The expression level of FTO in melanoma increases, and the body, through autophagy and the nuclear factor κ B pathway, induces FTO expression and affects m^6^A modification and demethylase activity, thereby regulating the expression of its target gene programmed death protein 1 (PD-1) and promoting the occurrence and development of tumours^22^. Recently, plant extracts, including resveratrol and curcumin, have been investigated for their ability to regulate m^6^A methylation 23, 24. However, the m^6^A regulation of AR extracts in melanoma remains unclear.

In this study, we aimed to explore the potential protective effects of AR extracts in melanoma therapy. Our primary objective was to understand whether these observed effects could be attributed to the modulation of m6A mRNA methylation, a critical process in melanoma development and progression. By examining the influence of AR extracts on m6A mRNA methylation, we aimed to identify potential therapeutic targets and provide mechanistic insight into the antimelanoma properties of these extracts. These findings could pave the way for the development of novel treatments for melanoma via the use of a plant extract of AR.

## Materials and Methods

### Animals

In this study, 4-6-week-old BALB/c nude mice were selected to construct a melanoma xenograft tumour model^25^. All the mice were fed under artificial light at 25 °C for 12 hours/day. Sixteen mice were randomly divided into a control group and an administration group, with 8 mice in each group. Control group: Melan B16 cells were injected subcutaneously to establish a mouse melanoma subcutaneous transplant tumour model. Administration group: After 8 days of successful modelling, the drug was administered via a 400 mg/kg *AR* extract orally once a day for 4 weeks. The mice were killed after intragastric administration, and the tumour tissues were collected for subsequent experiments. Detection: After 8 days of xenotransplantation, the mice in the control, model and experimental treatment groups were treated with the *Anoectochilus roxburghii* extract. After 14 days of treatment with the *Anoectochilus roxburghii* extract, the tumour tissues in the control, model and experimental treatment groups were collected and photographed, and the tumour volume was calculated. The animal study was reviewed and approved by the ethics committee of the School of Medicine, Tongji University.

### Cell culture and grouping

After being resuscitated, the cells were grown in a constant-temperature incubator (5% CO_2_, 37 °C) in foetal bovine serum medium containing antiserum. The experiments included an overexpression group, an interference group, an NC group and a blank control group, and each group had 3 replicates.

### Cell proliferation detection via the CCK8 method

The cells were divided into groups of 1 × 10^4^and inoculated into 96-well plates. After the mixture was shaken, it was placed in an incubator at 37 °C, 5% CO_2_ and saturated humidity. Transfection was carried out with 5 μg/μl of CCK-8 solution after 4 hours of incubation. A growth curve was generated with the cell growth time as the abscissa and the growth inhibition rate as the ordinate.

### Total RNA extraction and qPCR

After tissue homogenization, 1 ml of TRIzol reagent was added. cDNA was produced via reverse transcription via a one-step PrimeScript cDNA synthesis kit according to the manufacturer's instructions. Using this cDNA as a template, qPCR was performed using a one-step SYBR PrimeScript RT‒PCR kit. Target genes (*METTL3*, *METTL14, WTAP, Kiaa1429, FTO, and ALKBH5*) were expressed with *GAPDH* as an internal parameter, and the quantitative results were measured with 2- ΔΔ CT. The primers used are listed in Table 1.

### To construct siRNA cells

The interference sequence was designed and synthesized. Vectors were obtained via agarose gel identification, gel cutting and recovery. After enzyme digestion, the vector and gene were ligated, infected with Stbl3 competent cells, shaken and mixed, subjected to heat shock and an ice and water bath, and then incubated in nonresistant LB liquid medium for PCR amplification and sequencing identification. In accordance with the instructions of Lipofectamine 2000, the target plasmid was transfected into 293T cells, the transfection results were assessed via qRT‒PCR, the cell supernatant rich in lentiviral particles was collected, the virus was concentrated, and the virus titre was measured and calibrated in 293T cells. The lentivirus was transfected into target cells, which were verified via RT‒PCR.

### To construct lvrna cells

The designed and synthesized target gene sequence was ligated to the pcDNA3.1 vector. The purified and recovered target fragments were digested with restriction endonucleases to recover the enzyme fragments and then linked with the vector to synthesize the target gene recombinant plasmid. Then, the target gene recombinant plasmid and the pcDNA3.1 empty vector were transformed into DH5α competent *E. coli* cells. The resulting plasmids were extracted and sequenced for identification. In accordance with the instructions of Lipofectamine 2000, the target plasmid was transfected into 293T cells, the transfection results were detected via qRT‒PCR, the cell supernatant rich in lentiviral particles was collected, the virus was concentrated, and the virus titre was measured and calibrated in 293T cells. The lentivirus was transfected into target cells, which were verified via RT‒PCR.

### LC‒MS was used to determine the m6A level

The nucleic acid was broken into nucleosides and bases by combining the steps of enzyme treatment, dissociation and fragmentation. Two examples of m6A and total A were calculated from the mass spectrum and peak intensity.

### MeIP PCR detection

RNA fragmentation: After RNA extraction, 450 ml of IP buffer and 2 μLofRNase inhibitor were added to the sample to be tested, and a noncontact fully automatic ultrasonic crusher was used for RNA fragmentation. The parameters were set to high power (30 s+30 s), and 15-30 cycles were used for ultrasonication. The size of the segmented RNA was detected by 1% agarose gel electrophoresis. The 50-μL sample to be tested was used as the input group and stored at -80 °C until use. A 400-μL sample to be tested was used as the IP group. For immunoprecipitation, 20 μL of IP buffer 2 and 4 μgof m6A antibody or IgG were added to the IP group, mixed well, put into a vertical mixer, and allowed to react at 4 °C for 4 h.For the protein A/G magnetic bead treatment, the mixture was vortexed for 1 min to fully suspend the magnetic beads, and 25-50 μLof magnetic bead suspension was transferred to a 1.5-ml centrifuge tube. Two hundred microlitres of the buffer mixture was combined, and the centrifuge tube was placed on a magnetic rack and allowed to stand for 1 min, after which the supernatant was discarded after the mixture was clarified. The previous step was repeated twice for a total of three times. For protein A/G binding to the antibody, prepared protein A/G magnetic beads were added to the IP group, which was subsequently incubated in a vertical mixer at 4 °Cfor 1 magnetic beads were adsorbed on the magnetic frame to remove the supernatant. Then, 200 μL of wash buffer solution was added to the EP pipe, which was gently mixed, and the mixture was washed at 4 °C in a vertical mixer for 5 min. Magnetic beads were adsorbed on the magnetic frame to remove the supernatant. The previous step was repeated 2 times. Two hundred microlitres of buffer was added, and the RNA was extracted together with the input sample. For RNA detection and validation, an equal volume of RNA was used for reverse transcription and qPCR validation.

### RNA stability assay

The cells were plated with 5 μg/ml actinomycin D (Sigma) at the indicated times. The RNA was isolated, and the stability of the indicated RNA was determined via qRT‒qPCR.

### Statistical analysis

The measurement data conforming to a normal distribution are presented as the means ± SDs, independent samples were compared via t tests, and figures were generated with Prism 6. P < 0.05 was used to indicate statistically significant differences.

## Results

### AR extracts inhibit the growth of melanoma in a mouse subcutaneous xenograft tumour model

**Figure 1:**
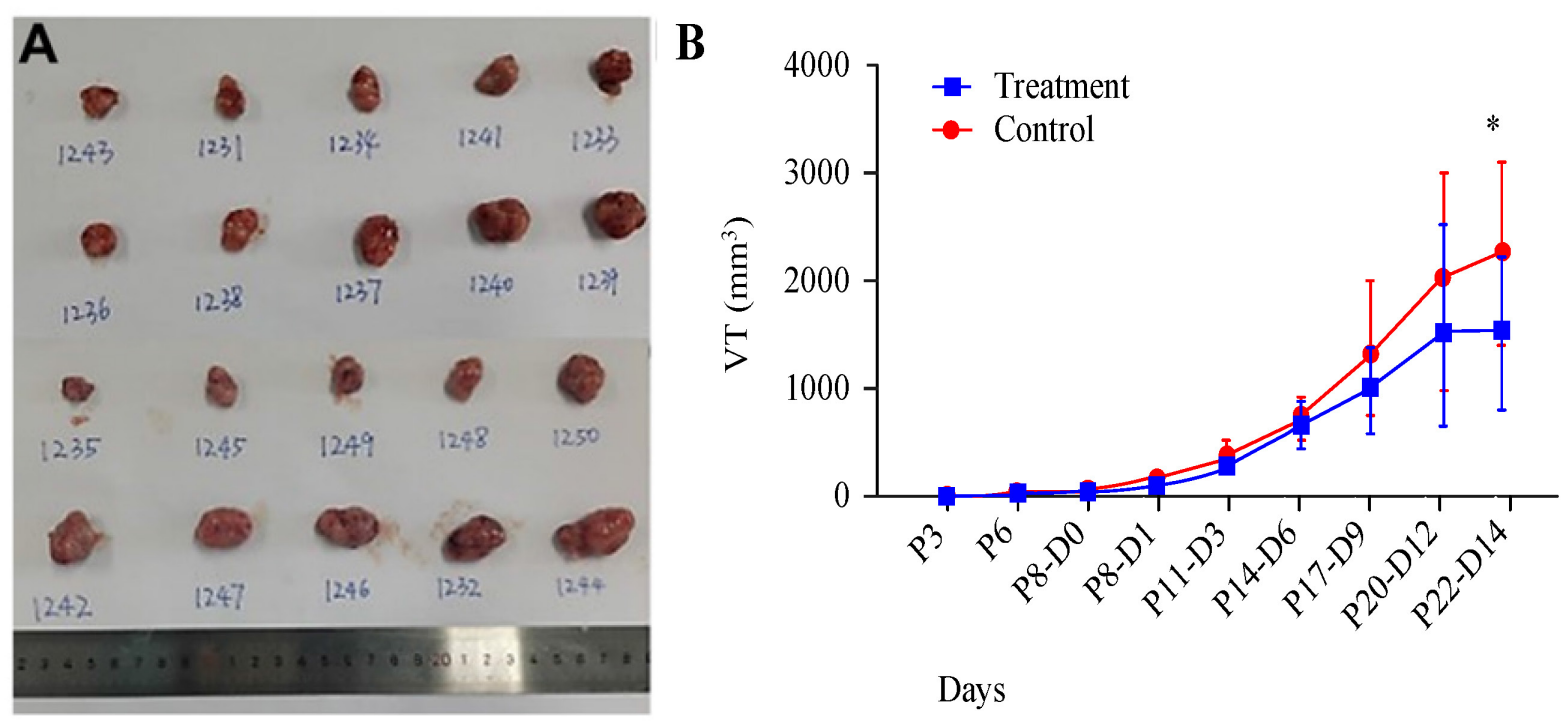
***Anoectochilus roxburghii* extracts inhibit the growth of melanoma in a mouse subcutaneous xenograft tumour model.** (A) This panel shows an image of a tumour extracted from a mouse, which visually captures the effects of the treatment. (B) This graph presents the measured tumour sizes over time, illustrating the change in tumour volume. Importantly, treatment with *Anoectochilus roxburghii* extracts commenced 8 days after the xenograft was established. An asterisk (*) indicates a statistically significant difference with a p value of less than 0.05, indicating that the observed inhibition of tumour growth is unlikely to be due to chance.

We utilized a subcutaneous tumour model in mice to evaluate the impact of AR extracts on melanoma growth. Observations indicated that, at 14 days posttreatment with AR extracts, the volume of the melanoma had been significantly reduced by approximately 75% compared with that in the control group (Figure 1A and 1B).

### AR extracts inhibit the expression of the RNA m^6^Amethyltransferase METTL3 in melanoma

**Figure 2:**
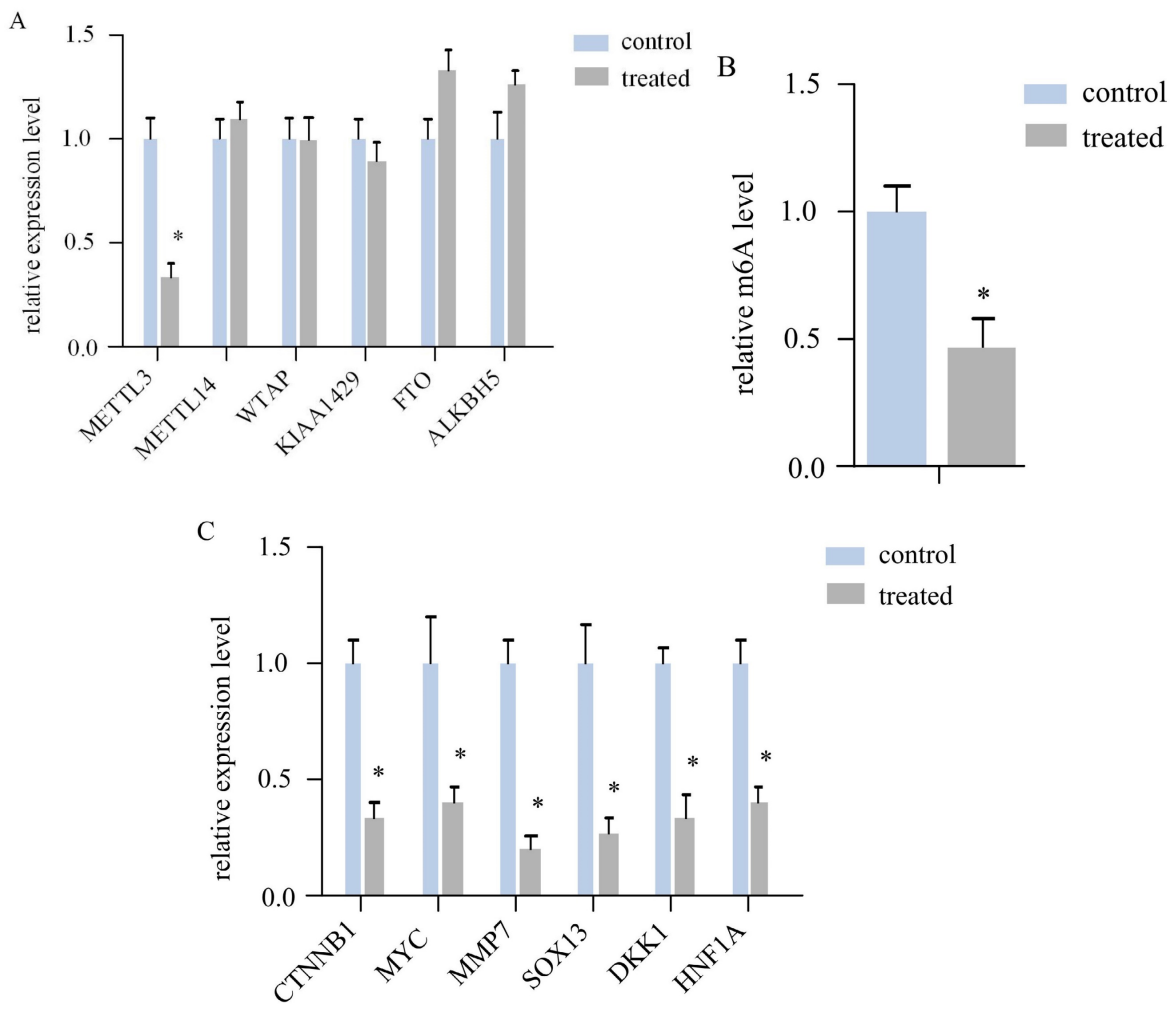
***Anoectochilus roxburghii* extracts inhibit the expression of *METTL3* and the WNT pathway in melanoma.** (A) This figure quantitatively depicts the gene expression levels of various RNA m6A modification regulators. Among them, METTL3 was significantly different, suggesting that *Anoectochilus roxburghii* extracts may specifically downregulate METTL3 expression. (B) Here, we compared the total RNA m^6A levels in melanoma cells, highlighting the impact of the extract on these epigenetic markers. (C) The diagram illustrates the changes in the expression levels of genes associated with the WNT signalling pathway, a critical pathway implicated in the regulation of cell proliferation and differentiation. An asterisk (*) indicates statistical significance with a p value of less than 0.05, underscoring that the changes in gene expression are non-random and could be attributed to the effects of the *Anoectochilus roxburghii* extracts.

To investigate whether AR extracts influence RNA m6A in melanoma, we assessed the expression levels of six crucial regulatory genes related to RNA m6A methylation. In a mouse subcutaneous transplant tumour model, melanoma tissue was harvested, RNA was extractedand converted to cDNA, and the target gene was then quantitatively amplified. The findings revealed that the mRNA level of *METTL3* in the AR extract treatment group was more than double that in the control group, corresponding to a statistically significant difference (Figure 2A). However, other regulatory components, such as *METTL14*, *WTAP*, *KIAA1429*, and other methyltransferase constituents, were not significantly altered. No difference was observed in the m6A demethylases*FTO*and *ALKBH5*, among others.

We subsequently measured the m6A modification level of the total RNA of both sample groups via LC‒MS. The AR extract treatment group was significantly downregulated by more thantwofold (Figure 2B). This evidence suggests that AR extracts may reduce the m6A modification level of RNA in melanoma by lowering the level of *METTL3*.

### Inhibition of Wnt/β-catenin signallinggenes by AR extracts in melanoma

According to the literature, *METTL3* can regulate *Wnt* signallingpathway genes to affect the proliferation of melanoma cells. The expression of *CTNNB1*, *MYC*, *MMP7*, *SOX13*, *DKK1*, and *HNF1A*, a total of 6 key genes of the Wnt/β-cateninsignalling pathway, was determined via quantitative PCR. The results indicated that all six genes were downregulated in the melanoma tissues of the AR extract treatment group (Figure 2C).

### By inhibiting METTL3, AR extracts inhibit melanoma cell invasion and migration

**Figure 3:**
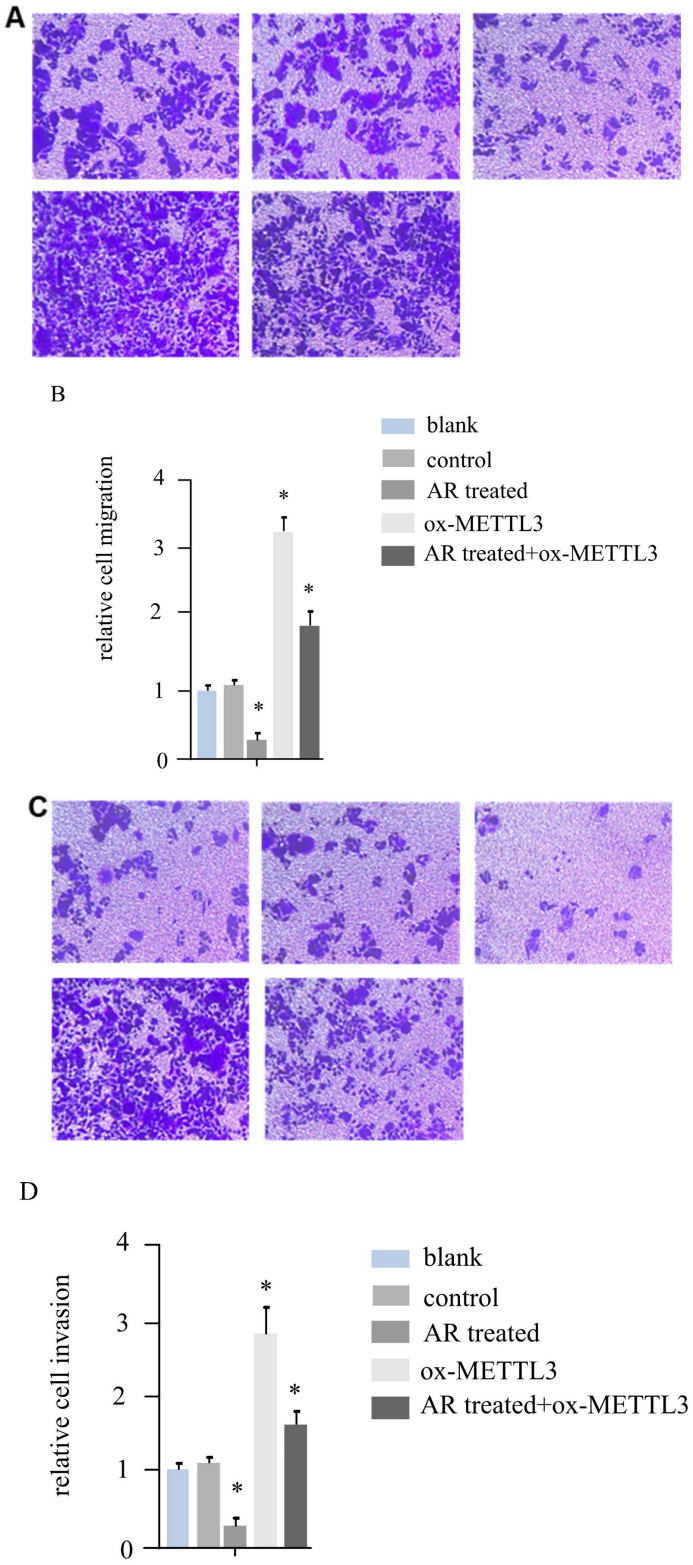
** Melanoma cell invasion and migration were inhibited by *Anoectochilus roxburghii* extracts through the downregulation of *METTL3*.** (A and B) These panels display the results of the cell invasion assays, where a marked decrease in the invasive capacity of melanoma cells was observed following treatment with *Anoectochilus roxburghii* extracts. Together, the images and quantitative data suggest a significant reduction in the number of cells able to penetrate the matrix barrier, which is indicative of the extract's inhibitory effect on cell invasion. (C and D) These figures illustrate the results of the cell migration assays, with both the visual representation and quantitative analysis demonstrating a noticeable decrease in the migration rate of melanoma cells after treatment. This reduction indicates that *Anoectochilus roxburghii* extracts effectively suppress the ability of cells to move and potentially spread, which is a critical aspect of cancer progression. An asterisk (*) denotes a p value less than 0.05, indicating that the observed decrease in invasion and migration was statistically significant and can be attributed to the effects of the *Anoectochilus roxburghii* extracts.

According to the literature, *METTL3* can promote the invasion and migration ability of melanoma cancer cells. For hs294t melanoma cells, in addition to treatment with AR extracts, *METTL3* was overexpressed, and a recovery experiment was performed in the AR extract treatment group. We constructed five groups of cells, namely, the blank group, control group, AR treatment group, *METTL3* overexpression group, and AR treatment + ox-*METTL3* overexpression group, and compared the migration and invasion abilities of the four groups of cells. The results revealed that the invasive ability of the plants in the AR treatment group was reduced by more than half compared with that of the control group, and the overexpression of *METTL3* was more than three times greater than that in the control group. However, it was significantly lower in the AR treatment + ox-*METTL3* group than in the ox-*METTL3* group (Figure 3A and 3B). The change in cell migration ability was similar to that in invasion ability among the groups. Compared with those in the control group, the number of METTL3-overexpressing cells in the treatment group was more than half that in the control group, and the number of METTL3-overexpressing cells was more than three times greater than that in the control group. The difference was statistically significant. However, it was significantly lower in the AR treatment + ox-*METTL3* group than in the ox-*METTL3* group (Figure 3C and 3D).

### In melanoma cells, AR extracts downregulated the expression of Wnt signalling genes by inhibiting METTL3

**Figure 4:**
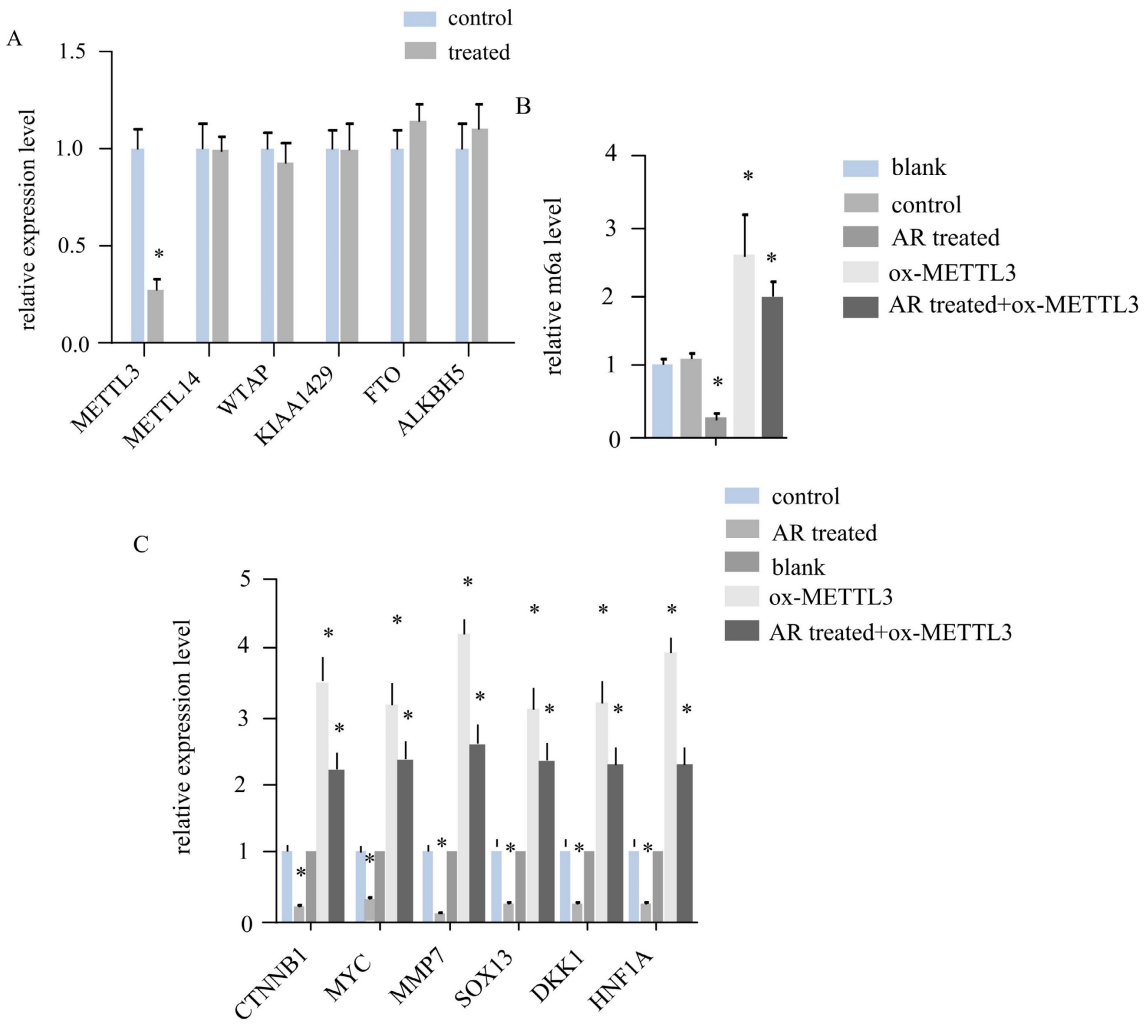
***Anoectochilus roxburghii* extracts inhibit the expression of the WNT pathway through downregulating METTL3 in HS294T.**(A) This panel details the gene expression levels of various RNA m6A regulators in the cells. The data presented here show that, of all the regulators assessed, the expression of METTL3 is notably decreased, implying a targeted inhibitory effect of the *Anoectochilus roxburghii* extracts on this specific regulator. (B) This portion of the figure quantifies the total RNA m6A modification levels, providing evidence of the overall reduction in epigenetic markers associated with m6A modification in response to the extract treatment. This graph depicts the expression levels of genes within the WNT signalling pathway, a crucial cascade in cellular communication that influences cell fate and behaviour. Substantial downregulation of these genes was observed, indicating that *Anoectochilus roxburghii* extracts may exert their anticancer effects by modulating this pathway. An asterisk (*) signifies a p value of less than 0.05, indicating that the reduction in both METTL3 expression and WNT pathway gene expression was statistically significant and suggesting a potential causal relationship between the effects of the *Anoectochilus roxburghii* extracts and the observed molecular changes.

In melanoma tumour tissues, AR extracts regulate the expression of *METTL3*, affect the level of RNA m^6^A and downregulate the expression of genes associated with the Wnt signalling pathway. By detecting gene expression in the five groups of cells in the blank group, control group, AR treatment group, ox-METTL3 group, and AR treatment + ox-*METTL3* group, we found that the expression of mettl3 in the AR treatment group was reduced by more than half that in the control group and that the expression levels of other *METTL14, WTAP, KIAA429, FTO*, and *ALKBH5*genes remained unchanged (Figure 4A). Compared with that in the control group, the level of RNA m6A was reduced by more than half in the AR treatment group and increased by more than twofold in the *METTL3* overexpression group, whereas the level of RNA m6A in the AR treatment + ox-*METTL3* group was much lower than that in the ox-*METTL3* group (Figure 4B). The mRNA levels of the key genes of the catenin signalling pathway exhibited the same trend among the groups. Compared with that in the control group, the expression in the AR treatment group was lower, and that in the overexpression group was greater; however, the expression in the AR treatment + ox-*METTL3*group was significantly lower than that in the METTL3 overexpression group (Figure 4C).

### AR regulates the RNA m6A level of UCK2 through METTL3

**Figure 5:**
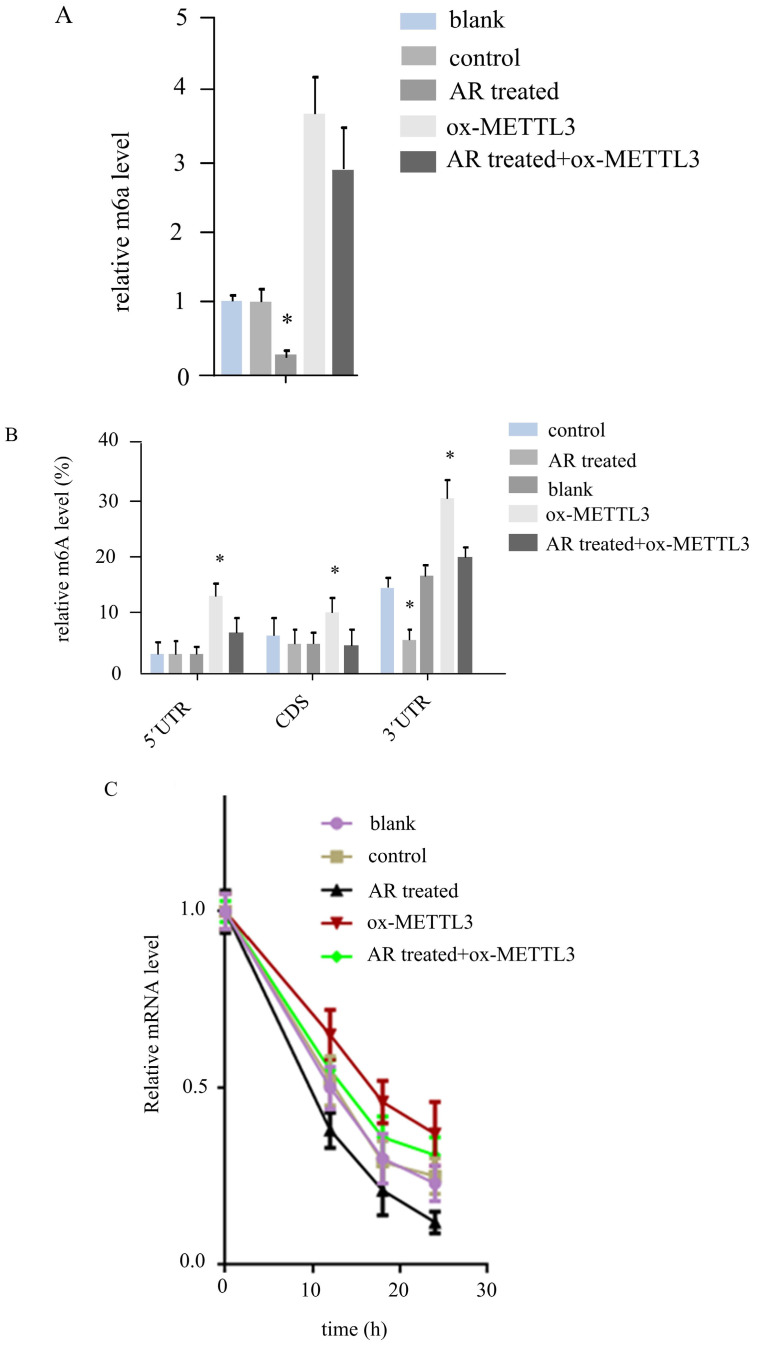
***Anoectochilus roxburghii* extracts inhibit UCK2 m6A by downregulating METTL3 in HS294T.** (A) The expression level of UCK2 significantly decreased as a result of treatment with *Anoectochilus roxburghii* extracts, which suggests a specific downregulation effect of the extracts on this gene. (B) This part of the figure illustrates the m6A modification levels at different regions of the UCK2 mRNA. The graphical representation clearly indicates a decrease in m6A modifications across these regions, which points to the influence of the extracts on the mRNA methylation landscape. The results of the mRNA degradation assay for UCK2 depicted here indicate the stability of the UCK2 mRNA transcripts. These results indicate an accelerated degradation rate in the presence of *Anoectochilus roxburghii* extracts, suggesting that the downregulation of METTL3 may lead to a reduction in mRNA stability and, thus, a decrease in UCK2 expression. This comprehensive analysis demonstrated the potential mechanism by which *Anoectochilus roxburghii* extracts may inhibit melanoma cell proliferation and survival through an intricate interplay with epigenetic regulatory mechanisms.

*METTL3* has been reported to affect Wnt signalling by regulating the m6A level of* UCK2*/β-catenin signalling genes. We determined the mRNA level of *UCK2* in the above five groups of cells. The results revealed that *UCK2* was downregulated by half in the AR treatment group and upregulated 3-fold in the ox-*METTL3* group, whereas the expression of *UCK2* was downregulated in the AR treatment group and the mettl3-overexpressing group compared with the ox-*METTL3* group. These differences were statistically significant (Figure 5A).

By MeIPqPCR, we detected m6A changes in different regions of the UCK2 mRNA in the five groups of cells. In the 5'UTR and CD regions, AR treatment did not affect the m6A level, and the m6A level in the ox-*METTL3* group was greater than that in the control group, while that in the AR treatment + ox-*METTL3* group was lower than that in the ox-*METTL3* group. In the 3'UTR region, the level of m6A decreased in the AR-treated group compared with the control group and was increased in the ox-*METTL3* group, whereas the level of m6A decreased in the AR+ ox-*METTL3* group compared with the ox-*METTL3* group (Figure 5B).

Furthermore, we detected the degradation rate of *UCK2* mRNA in the five groups of cells via RNA degradation rate experiments. The results revealed that the rates of *UCK2*degradation decreased in the AR treatment group, control group, AR treatment + ox-*METTL3*group, and ox-*METTL3* group (Figure 5C).

## Discussion

Melanoma is still a very dangerous tumour, and its incidence rate is increasing annually. Advanced melanoma is still difficult to cure^4^, ^7^. The most obvious feature of this type of tumour is its highly invasive and expansionary growth^1^. Therefore, research has paid special attention to the growth and invasion of melanoma. In this study, AR extract significantly reduced tumour growth and inhibited the invasion and migration of melanoma cells in vitro. Although many plant extracts have been found to have antitumour, antioxidant and immunomodulatory effects on melanoma treatment, the results still provide a basic concept for exploring the regulatory effects of natural products on melanoma and provide new ideas for prevention and treatment^8^-^10^. AR is widely distributed throughout Asia and has been cultivated artificially. It is a rich drug resource. AR extracts have been shown to have antitumour, immune stimulatory and antihyperglycaemic functions^12^-^14^. However, no study has reported the relationship between it and melanoma migration. In this study, its inhibitory effect on melanoma was demonstrated via in vivo and in vitro experiments, and one of its possible regulatory pathways was preliminarily explored.

m^6^A modifications are important epigenetic modifications that have been studied in recent years. Among them, the m^6^A methyltransferase complex, demethylases and m6A binding proteins have been confirmed to play critical roles in the occurrence and development of various tumours^16^, ^26^. The role of RNA m^6^A in melanoma has been confirmed, and some reports have analysed the mechanism by which mettl3 regulates melanoma cell migration^20^, ^21^. In this study, both in vitro and in vivo experiments demonstrated that AR extract could downregulate *METTL3* in cancer cells and reduce the m6A modification level of total RNA. Moreover, AR extract can reduce the m6A level of the key* UCK2* in melanoma cell migration and reduce its mRNA level. Furthermore, we confirmed that AR extract affects the expression of *UCK*2 by regulating *METTL3*. Consistent with these reports, we found that these effects lead to differences in the expression of Wnt signalling pathway genes and ultimately affect the migration and invasion ability of cancer cells. In addition, through RNA degradation rate experiments, we found that the AR extract can regulate the stability of its mRNA by affecting the m6A level of *UCK2*, thereby affecting gene expression. Several recent studies have explored the role of RNA m6A modification in various cancers, demonstrating its importance in cancer biology. METTL3, a critical m6A methyltransferase, has been implicated in the progression of numerous cancers, including melanoma. One such study revealed that METTL3 promotes the growth and mobility of melanoma cells^27^. These results are in accordance with those of the present study, as they also demonstrate the significance of METTL3 in melanoma cell migration. Another relevant study revealed that METTL3 regulates the expression of oncogenic MYC in acute myeloid leukaemia through m6A modification^28^. These findings further reveal the role of METTL3 as a regulator of gene expression in cancer cells. Additionally, a study highlighted the role of m6A modification in the Wnt signalling pathway, where it accelerates colorectal carcinogenesis^29^. This finding is particularly interesting, as our study also revealed changes in Wnt signalling pathway gene expression resulting from AR extract treatment. These findings align with our findings on the ability of AR extract to regulate UCK2 mRNA stability through m6A modification. In summary, these studies provide evidence that AR extract can influence m6A modification and, consequently, cell migration and invasion in melanoma. These findings also highlight the significant role of METTL3 and m6A modifications in cancer biology, further highlighting the potential of AR extracts as therapeutic agents.

In conclusion, we found that AR extract can reduce the expression of *METTL3*, reduce the RNA m6A modification of *UCK2*, accelerate the degradation of UCK2, reduce the expression of UCK2, and thereby downregulate the Wnt signalling pathway, which is one of the key mechanisms involved in weakening the invasion and migration of melanoma cells and inhibiting the growth of tumours.

## Figures and Tables

**Table 1:** Primer list

GENE	P1(5' to 3')	P2
METTL3	ACAACAGAGCAAGAAGGTCAGTCAG	TTCATGCACTCCTCCTTGGTTC
METTL14	AGGGATGTAGGTTTAGCTG	GTTCTCTGATGTCAAAGGC
METTL16	TCGTTGTCACGACATGGATTGA	CACACGCTCTCTTTTCTTTCTCCTT
WTAP	ATGACCAACGAAGAACCTCTTC	GCCCTCCAAAGCTTGTACATAT
KIAA1429	AAGTGCCCCTGTTTTCGATAG	ACCAGACCATCAGTATTCACCT
FTO	CTGAGGCTTCTTGAAGAGCTTGAA	TGAACCTCTTTATGGAGCTCCTCA
ALKBH5	GCCAGCGGCTACACGGACCTG	AAGGTTCGGCGGCAGCGG
